# Annotations

**Published:** 1898-03-19

**Authors:** 


### ANNOTATIONS.

### London Air.

The first number of the "Transactions of the British Institute of Preventive Medicine" contains, in an article by MacFadyn and Lant, some interesting statistics relating to the presence of dust and bacteria in London air. In a suburb the dust particles numbered 20,000 per cubic centimetre in the open air, and 44,000 in a quiet room; in the City, on the other hand, the totals per cubic centimetre were 500,000 when taken from a roof, 300,000 in a yard, and about 400,000 in a room. Causes apparently small gave rise to great variations in the number of dust particles; thus, out of doors a puff of wind would increase them to such an extent as to render counting impossible, whilst in rooms any sudden movement of persons or furniture, or the smoke from a cigarette, would cause a marked rise. In the City the dust contamination was 900 per cent. greater than in the suburbs, which fact is in accord with the general experience that fogs are both more dense and more frequent over the centre than in the outskirts of the City. Although dust is the great carrier of micro-organisms, the proportion of the one to the other was found to be not higher than 1 organism to 38,000,000 particles. Calculating 100 organisms per cubic metre, a man would, in the course of his three score years and ten, inspire about 25,000,000 microbes, which is about the number contained in half-a-pint of milk. These figures illustrate very forcibly the extent to which our atmosphere is polluted by chimney smoke and street dust. The proportion of micro-organisms to dust particles, and, indeed, the number actually present in air, however, is strikingly low. About one-half of the living forms proved to be moulds, so that the chance of a wound becoming infected from the air must be extremely small. Aërial disinfection by carbolic spray is now practically obsolete, and the present data tend to accentuate the necessity for attention being given to sterilisation of instruments and hands, and the abolition of all dust-traps in surgical wards rather than to protection from the air.

### Well Done, "Pick-Me-Up"!

The case of Owen v. Greenberg, which came before the Court of Queen's Bench last week, is one of very considerable importance from the point of view of general morality, for by the verdict given it is decided that many of the advertisements which so often appear in newspapers in regard to medicines purporting to remove various female obstructions are of an immoral nature, notwithstanding that they may not contain any specific statement as to the condition the medicines are intended to relieve. The action was one brought by Mr. Edward Owen, trading as Frederick Allen, of 145, Stockwell Road, Surrey, against Messrs. Greenberg and Co., of 80, Chancery Lane, to recover damages for breach of contract by the defendants to insert certain advertisements in the journal *Pick-Me-Up* relating to medicines for ladies. The defence was that the defendants were merely agents for the proprietors of *Pick-Me-Up*, and were therefore not personally liable, and, further, that the advertisements were of an immoral and illegal nature, and that the plaintiff therefore was not entitled to maintain any action in respect thereof. It appeared that the plaintiff carried on business at 145, Stockwell Road, as Dr. Allen, U.S., and as the Irristum Company, and at Clapham as the Continental Drug Company. The plaintiff had paid the defendants in advance the price of the insertion of the advertisements. The advertisements appeared in *Pick-Me-Up* for some time, but upon a change of proprietorship the new proprietors declined to continue to insert them. Dr. D. H. Attfield, Bachelor of Surgery, Bachelor of Medicine, M.A., and who also holds a diploma of health from the University of Cambridge, said that with the first bottle, which cost 4s. 6d., was a notice that if the bottle was not quite enough to effect a cure a second should be written for. With the second a notice that if not strong enough a stronger bottle at 10s. should be obtained, and with the third bottle a notice that a still stronger bottle at 21s. should be obtained. There was no difference in the contents of the bottles upon analysis. The jury found that the defendants acted as principals, that the amount in their hands was £96 15s. 6d., that the plaintiff sustained no damage, and that the contracts for insertion of the advertisements were immoral. His lordship (Mr. Justice Darling) entered a verdict and judgment for the defendants, and made an order for payment to them of £101 11s. 7d., the amount which they had paid into Court. Evidence was given in the course of the trial that similar advertisements appeared in any number of respectable papers; in fact, that they were scattered broadcast through the Press. We hope that after this definite pronouncement as to their character such advertisements will be looked on a little more shyly by newspaper managers.

### The "Star System" in Medical Societies.

A system appears to be taking root in the State of Pennsylvania which, if it extends, may perchance effect changes in the medical world and in the meetings of medical societies, analogous to those which fell upon provincial theatres when touring "stars" supplanted the old stock companies. It seems that the Medical Society of the State of Pennsylvania issues a list of physicians and teachers who are willing to lecture or hold clinics upon given subjects before county societies throughout the State, and it is claimed that thus men in outlying parts will have an opportunity of hearing the views of specialists and teachers of repute, and of so increasing their knowledge of the most recent discoveries and suggestions in medical practice. This is all very well, but it means destruction to that mutual interchange of thought and experience which used to be, and still to some extent is, characteristic of the meetings of the most useful of our medical societies. To sit and listen to a great star lecturing on his own specialty, and then to go home again, may be very improving; but for keeping up the tone and character of medical work in a district there is nothing like mutual comparisons of personal experiences, and we should be sorry to think of our provincial societies degenerating into mere lecture shops of the University Extension type. So far this canker, except in a few isolated instances, has only attacked the "introductories," but it is difficult to see where it might end if encouraged.

